# Whole-Genome Sequence of the *Wolbachia* Strain *w*Tcon, an Endosymbiont of the Confused Flour Beetle, Tribolium confusum

**DOI:** 10.1128/mra.01144-21

**Published:** 2022-02-17

**Authors:** Yeganeh Gharabigloozare, Alexander Wähling, Christoph Bleidorn

**Affiliations:** a Department of Animal Evolution and Biodiversity, Johann-Friedrich-Blumenbach Institute for Zoology and Anthropology, University of Göttingen, Göttingen, Germany; b Institute of Computer Science, Campus Institute of Data Science, University of Göttingen, Göttingen, Germany; University of Maryland School of Medicine

## Abstract

Up to 60% of insects are infected with symbiont intracellular alphaproteobacteria of the genus *Wolbachia*, which are often able to manipulate their host’s reproduction. Here, we report the annotated draft genome sequence of strain *w*Tcon from the confused flour beetle, Tribolium confusum, based on long- and short-read sequence data. The assembled genome is located on 12 contigs with a total size of 1,418,452 bp.

## ANNOUNCEMENT

Tenebrionid beetles of the genus *Tribolium* include cosmopolitan pests of many stored products, from flour to dried foods (1). Due to minimal maintenance work and access to all life stages, two different tenebrionid beetle species, the red flour beetle (Tribolium castaneum) and the confused flour beetle (Tribolium confusum), have been studied in the lab for decades as model systems (2). *Tribolium confusum* is known to be naturally infected with *Wolbachia* (3–5), an intracellular alphaproteobacterium which has been found as a widespread endosymbiont of arthropods and nematodes (6). To support the utility of *Wolbachia* in the *Tribolium* model system, we report the draft genome sequence of *w*Tcon.

DNA was extracted from adult *T. confusum* beetles of the *Wolbachia*-infected strain MN61 (originally collected in Kansas, USA), supplied by the Stored Product Insect and Engineering Research Unit of USDA-ARS. Beetles were reared on flour medium and brewer’s yeast (5%) at 30°C and 65% relative humidity with a 16:8-h dark/light cycle. For Oxford Nanopore Technologies (ONT) long reads, high-molecular-weight (HMW) DNA from beetles (10 males and 10 females) was extracted using both a MagAttract HMW DNA kit (Qiagen, Hilden, Germany) and phenol-chloroform extraction with precipitation by sodium acetate (7). Two ONT libraries were prepared (SQK-LSK109)—without fragmentation or size selection—and sequenced using MinION (FLO-MIN 106) protocols from ONT. The raw ONT reads from two separate runs were base called using Guppy v3.4.4, yielding 4,074,131 reads with an *N*_50_ value of 2,511 bp. Furthermore, quality assessment and trimming were performed using FastQC v0.11.9 (8) and NanoFilt v2.8.0 (9), respectively. For short-read sequencing, genomic DNA was isolated using the Quick-DNA Miniprep Plus kit (Zymo Research, Irvine, CA, USA). Library preparation and sequencing were performed by AllGenetics (A Coruña, Spain), using Illumina paired-end (150-bp) libraries, sequenced using an Illumina HiSeq 2500 instrument, yielding 46,330,553 paired-end reads, which were trimmed using FastQC v0.11.8 (8). Default parameters were used except where otherwise noted.

The initial metagenome assembly for the ONT long reads was generated using Flye v2.8.1 (10), which estimated the size of the genome at around 1.4 Mbp. Using a BLAST search against the reference genome of the supergroup B *Wolbachia* strain *w*PipPel from Culex quinquefasciatus (GenBank accession number NC_010981.1), we identified putative *Wolbachia* contigs in the metagenome, and by mapping reads using Minimap2 v2.13-r850 (11) onto these contigs, we retrieved putative *Wolbachia* ONT raw reads. For Illumina reads, we created a metagenome assembly using SPAdes v3.13.2 with a *k*-mer of 77 (12). After mapping the trimmed raw reads onto this assembly using Bowtie v2.3.4.1 (13), we were able to extract short reads of putative *Wolbachia* origin.

The hybrid assembly of *w*Tcon using the extracted long and short reads was completed using Unicycler v0.4.9 (14), which contains 12 contigs (*N*_50_ value, 138,551 bp) in a total length of 1,418,452 bp (GC content, 34.1%). The draft genome was annotated by the NCBI Prokaryotic Genome Annotation Pipeline (PGAP) (15). The annotation identified 1,236 protein-coding genes, 58 pseudogenes, 34 tRNAs, 4 noncoding RNAs (ncRNAs), and 3 rRNAs (5S, 16S, and 23S). We assessed the genome completeness using BUSCO v5.2.2 (16). Out of 364 searched BUSCO groups, 353 were complete and single copy, 2 complete and duplicated, 11 missing, and none fragmented, resulting in a 96.9% BUSCO completeness score. We compared the five housekeeping markers of the multilocus strain typing (MLST) system (17), namely, *gatB*, *coxA*, *hcpA*, *fbpA*, and *ftsZ*, with the public PubMLST database (https://pubmlst.org/) and could confirm that our sequenced strain shows the exact profile as available for *Tribolium confusum* (strain Tcon_B_BhAvill AK; *Wolbachia* sequence type 30 [ST30]).

### Data availability.

The accession number for the complete genome sequence of *w*Con in GenBank is JAIZNT000000000. In addition, the raw sequence reads for Oxford Nanopore and Illumina sequencing have been deposited in the NCBI Sequence Read Archive (SRA) under the BioProject accession number PRJNA767570.
